# Temporal modeling of nitrogen dioxide levels on Main Street, East Los Angeles: Estimating annual periodic components using the Variable Bandpass Periodic Block Bootstrap

**DOI:** 10.1371/journal.pone.0309790

**Published:** 2024-09-06

**Authors:** Megan Di Maio, Edward Valachovic

**Affiliations:** Department of Epidemiology and Biostatistics, School of Public Health, University at Albany, State University of New York, Albany, New York, United States of America; Federal University of ABC, BRAZIL

## Abstract

In this study we assess periodicities in nitrogen dioxide levels at a location in Los Angeles using a novel Variable Bandpass Periodic Block Bootstrap (VBPBB) method resulting in confidence interval bands for the periodic mean. Nitrogen dioxide (NO_2_) is an air pollutant primarily produced by the combustion of fossil fuels by power plants and vehicles with internal combustion engines which has been linked with a variety of adverse health outcomes including dementia, breast cancer, decreased cognitive function, increased susceptibility to Covid-19, cardiovascular and respiratory mortality. Previous analysis methods such as block bootstrapping can obscure periodically correlated patterns in time series. The sampling destroys the correlation observed in the data for patterns of different periods, such as the daily, weekly and yearly patterns of nitrogen dioxide levels we wish to investigate. We use the VBPBB method to isolate significant periodicities using a band pass filter before bootstrapping so that the correlations between the data are preserved. Confidence interval bands for VBPBB are compared against existing block bootstrapping. The resulting narrower confidence interval bands created by VBPBB show a significant annual fluctuation in nitrogen dioxide levels while the existing methods do not show it as clearly. Better characterization of pollution patterns will aid in pollution reduction efforts by allowing us to pinpoint times of highest risk and direct mitigation efforts where they will have the greatest impact. This technique exhibits potential for future applications to other areas of environmental and health interest and concern.

## Introduction

Nitrogen dioxide (NO_2_) is a pollutant with many documented harmful effects for human health. Although it can be naturally occurring, created by lightning strikes and arising from the soil, the great majority of NO_2_ is created by internal combustion engines and power plants that use fossil fuels [1]. NO_2_ has been linked with a variety of adverse health outcomes including dementia, breast cancer, decreased cognitive function, increased susceptibility to Covid-19, cardiovascular and respiratory mortality [2–8]. NO_2_ exposure has also been shown to disproportionately affect communities of color and economically disadvantaged communities [9]. The US Environmental Protection Agency monitors for NO_2_ and other oxides of nitrogen as part of the National Ambient Air Quality Standards and has set “a 1-hour standard at a level of 100 ppb based on the 3-year average of 98th percentile of the yearly distribution of 1-hour daily maximum concentrations, and an annual standard at a level of 53 ppb” [10]. A total of 896 sites throughout the country are currently monitored for compliance with the 2010 standard for NO_2_ [11]. We selected a site in Los Angeles for analysis since it has the most complete data available.

Nitrogen dioxide levels are affected by a variety of factors such as topology, wind, and traffic patterns. Winds around Los Angeles are shaped by larger patterns such as the Santa Ana winds as well as features such as mountains and canyons that interact with and shape the winds given its direction [12]. This in turn affects the levels of NO_2_ in the air. Addressing the impact of topology and winds on pollution is beyond the scope of this paper but may contribute to variability in the data observed. Car engines create NO_2_ pollution, and those patterns vary based on season, weekends, and holidays as well as throughout an individual day with expected peaks at rush hours. NO_2_ data is likely to reflect the complex interaction of these patterns. Understanding typical variations such as those by season better let us identify and potentially mitigate sources of pollution. It also lets us identify if a reading is particularly unusual if it occurs outside of the expected patterns of fluctuation. We can identify potential periodic patterns through visual examination and then by looking at spikes in the periodogram. If a measurement is periodic, it will be identifiable by frequencies of higher amplitude on the periodogram. Many traditional time series methods do not allow for the analysis of patterns with multiple periodicities. An appropriate method should permit determination of the existence and estimation of the effect of one or multiple periodicities within the time series.

Traditional bootstrapping methods such as those described by Efron and Kunsch take repeated independent resamples from a sample data set with identical size as the original sample [13, 14]. A statistic such as the mean is calculated for each resample, collectively forming an estimate of the sampling distribution of that statistic. Confidence intervals (CI) for the statistic can then be produced from the quantiles of the estimated sampling distribution. Bootstrapping is a powerful nonparametric technique however some complications arise when it is applied to time series data. In many data sets such as those collected through random sampling, observations are often independent of each other and so non-correlated. When observations are randomly selected in the bootstrapping process the location of the original points relative to each other is irrelevant since they are not related. However, time series data by its nature often contains data that is correlated with observations that are close to each other or occurring at periodic intervals more closely related. For example, when considering traffic data, the average number of cars on the road in January at 8 am is more closely related to the number of cars on the road in February at 8 am than in February at 3 am given human behavior and patterns of commuting. A standard bootstrapping technique does not consider these relationships and the samples are treated as if they were completely independent of each other when they are actually correlated. One approach to mitigate this problem is the General Seasonal Block Bootstrap (GSBB) proposed by Dudek [15]. GSBB preserves the principal component (PC) in resampling time series, however since GSBB bootstraps all components simultaneously the PC component of interest is preserved but noise, trend, and other PC components at different frequencies are included in the sample as well. This increases the variability of the data and makes larger and less precise CIs. In some cases, it can obscure the significance of a relationship.

The Variable Bandpass Periodic Block Bootstrap (VBPBB) method accounts for this by filtering the data before bootstrapping [16]. It uses the Kolomgorov-Zurbenko Fourier Transform (KZFT) band pass filter to take the bootstrapped samples from a smaller set of frequencies thus removing noise and preserving the correlation of the original data. It allows the data to be filtered for each principal component separately and thus better preserves the structure of the original data, allowing for more precise and accurate estimates [17]. These filters have been used in a variety of fields including modeling ozone, global temperatures, and diseases such as skin cancer, diabetes and COVID 19 [18].

In this manuscript we will perform a separate analysis on the NO_2_ time series data using the GSBB and VBPBB techniques and compare the resulting confidence bands for each of the selected principal components. We seek to determine if there are periodic patterns in the measured levels of NO_2_ and observe differences in the two methods.

## Methods

Air quality data readings were obtained from the United States Environmental Protection Agency’s Pre Generated Data files for January 2010 until June 30 of 2022 [19]. Measurements for nitrogen dioxide levels at the WGS84 datum point located in Los Angeles at longitude -118.22688 and latitude 34.06659 were selected and the hourly average of readings was taken.

**Fig 1:**
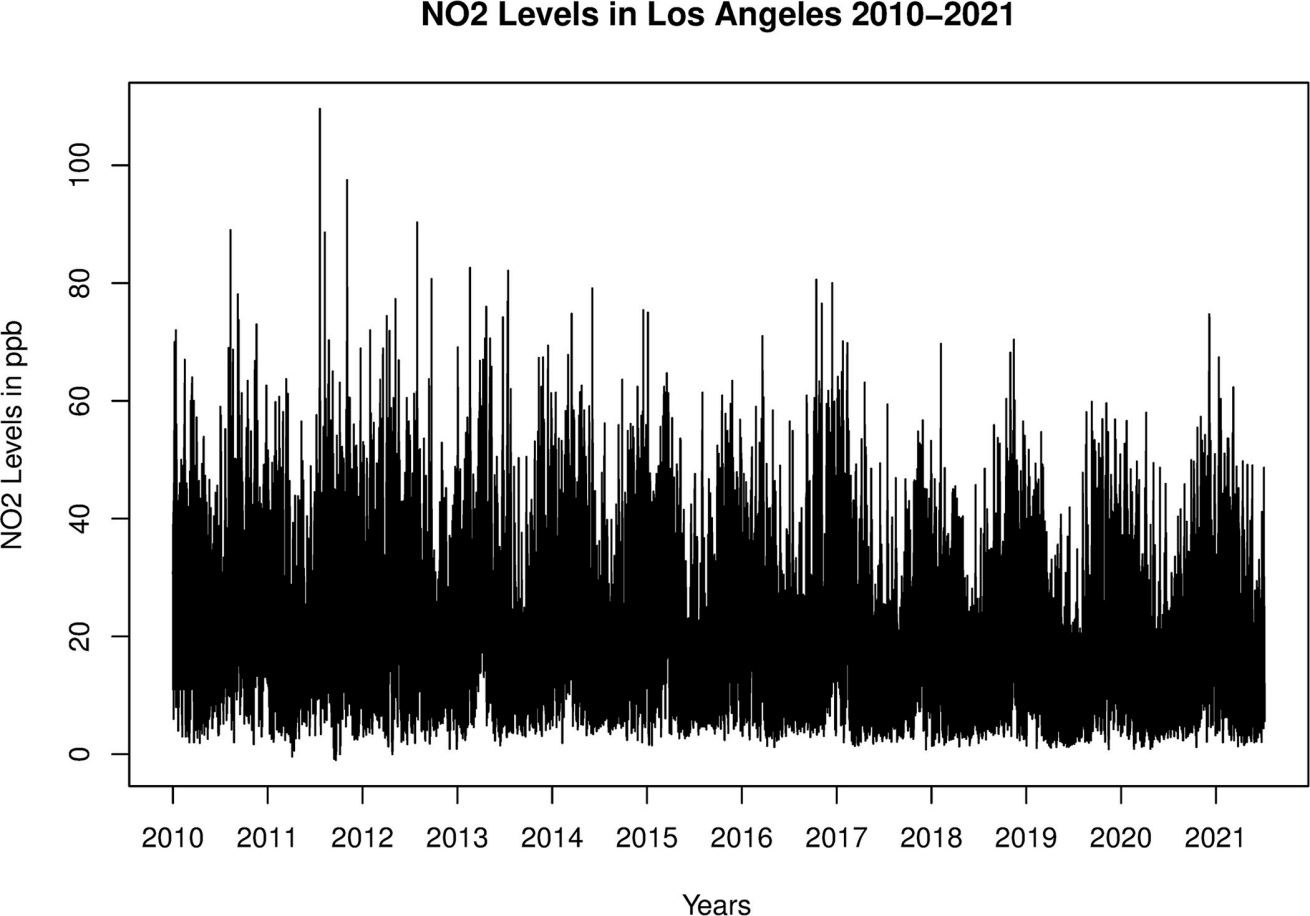
Hourly average levels of N0_2_ in ppb recorded in Los Angeles between 2010 and 2021.

There were 99,825 sample readings. Values had a median of 17.90 and a mean of 20.57 parts per billion. Coded errors in the original data account for only 5 readings out of the 99,825 considered. Fig 1 shows a time series of the hourly readings with apparent periodic fluctuation as well as substantial noise. There is a slight linear trend to the data showing a modest decrease in observed NO_2_ levels. It is unnecessary to detrend the data since the bandpass filters that we applied will largely suppress its impact since linear trends have a frequency distinct from the frequencies of the periodic components. All graphs were created by the author in R using base R and the KZA package.

**Fig 2:**
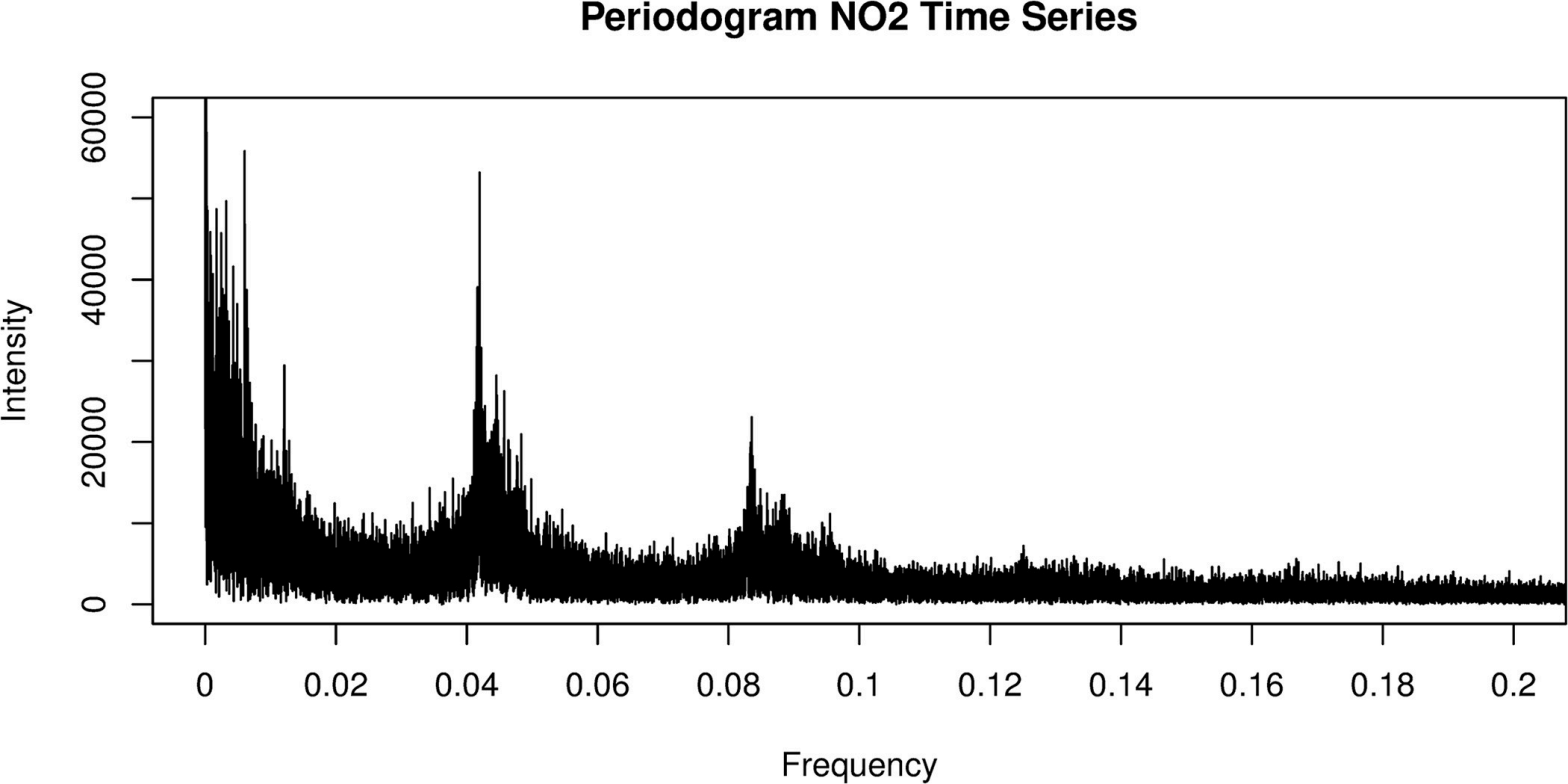
Periodogram of frequencies present in the NO_2_ time series.

Fig 2 shows the periodogram of the observed NO2 levels between January 2010 and June 30, 2021. A periodogram is a representation of the intensity of different frequencies present within the time series. Higher intensities represent frequencies which are more likely significant within the time series.

The periodogram showed notable peaks in values at 0.04 and 0.08, representing periods of 24 and 12 hours, however noise makes it difficult to identify other potentially common frequencies by inspection. We also tested annual, weekly, and daily components due to their strong connection to natural and human activity. Furthermore, a biweekly component was added upon inspection of the periodogram.

PC components at a given primary or fundamental frequency may not have a sinusoidal pattern but instead may exhibit a more complex pattern at the fundamental frequency. We can better fit the more complex pattern of the original data by modeling a complex wave as a sum of sinusoidal waves as in a Fourier transformation. A harmonic is an integer multiple of the fundamental frequency and is also periodic at that frequency and so is also useful in this analysis. Therefore, we investigate the fundamental frequencies along with some of their harmonics. Since we were investigating a pollutant which is a byproduct of internal combustion engines, we chose time intervals that are related to cycles of human movement. We included an annual component because of possible impact of weather including wind and temperature changes. Weekly was chosen because we anticipated differences in travel patterns between weekends and weekdays. Daily was selected since traffic levels fluctuate throughout the day. We also inspected the periodogram to look for other possible frequencies of note and included a second harmonic for the daily component based on our observations.

### Kolmogorov-Zurbenko Fourier Transform filter

Previous PC analysis methods such as block bootstrapping can obscure patterns in the time series since they can only account for one period length. The sampling will destroy the correlation observed in the data for patterns of different periods. Many real-world models contain multiple principal components, all with different periodicity, and so cannot be adequately replicated by the block bootstrapping method. For example, if there is a weekly pattern to the data taking blocks of length seven days will preserve the weekly pattern but may obscure a monthly or yearly pattern that also exits. In effect the other components will become noise after resampling and increase variability in the bootstrapped statistic [16].

Additionally, real-world data frequently contains trends, shifts, or simply noise, all of which have unique patterns of variation at different frequencies, and which can interfere with accurate modeling using bootstrapping. When data with these characteristics is bootstrapped, confidence intervals will be wider because of the increased variability these characteristics add to the data. By filtering to preserve a narrow band of frequencies VBPBB removes most of the interfering frequencies allowing for more precise estimation of the PC component sampling distribution.

The VBPBB uses Kolmgorov-Zurbenko Fourier Transform (KZFT) filters to pass a narrow band of frequencies surrounding a PC component frequency.

$${KZFT}_{m,k,\nu }\left(X(t)\right)=\sum_{u=-k(m-1)/2}^{k(m-1)/2}\frac{a_{u}^{m,k}}{m^{k}}e^{-i2m\nu u}X(t+u),$$ (1)

where the coefficients $a_{u}^{m,k}$ are the polynomial coefficients from:

$$\sum_{r=0}^{k(m-1)}z^{r}a_{r-k(m-1)/2}^{m,k}={\left(1+z+\cdots +z^{m-1}\right)}^{k}$$ (2)

Further information about the Kolmogorov-Zurbenko Fourier Transform can be found in Yang and Zurbenko’s 2010 article in WIREs Computational Statistics and in Valachovic’s recent work [16, 20].

Principal components were calculated using the KZA R package’s KZFT function to filter fundamental frequencies of interest including annual, weekly, daily and their harmonics.

In the R package, KZFT is a bandpass filter with three functional arguments including f, the frequency, m, the length of the moving average, and k, the number of times that the filter will be applied. The argument f is the central frequency of the bandpass filter and is set to the frequency λ, or the reciprocal of period 1/p, for the component of interest. The argument m is the length of the moving average. We wish to place the point at which the KZFT bandpass filter completely attenuates frequencies halfway between the desired frequencies to be separated. Because the equation requires an odd number, we added one to 2p, guaranteeing an odd result. Any odd number would have worked, but we chose the smallest one since we were trying to find the minimum value for m. As Valachovic describes in his recent article, “Periodically correlated time series and the Variable Bandpass Block Bootstrap”, increasing the width of the window m decreases the width of the bandpass filter, suppressing additional frequencies, resulting in a narrower bootstrapped confidence interval band for the periodic mean [16]. Larger moving average m values also create more time points where the filter is incompletely applied, since near each end of the data set the moving average exhausts available data.

The argument k represents the number of iterations for the filter, with a simple moving average for k = 1 and a nearly Gaussian curve for k = 3. Generally increasing k narrows the resultant energy transfer function and with our data tended to increase the amplitude of the calculated confidence bands. Since increasing k is more computationally intensive, we chose to keep k at 2 to increase efficiency. We are engaged in additional research to determine optimal arguments for analysis.

Block bootstrapping was performed on the original data set according to the GSBB. Block bootstrapping was performed on each of the frequency separated PC component data according to VBPBB. Then, 95% confidence interval bands for the periodic mean were calculated for each principal component to see if there was a significant variation in NO_2_ levels during the studied period. Confidence interval bands for VBPBB were then compared to those from GSBB to see if VBPBB led to narrower confidence intervals. All calculations were done in R version 4.3.2 using the KZA package and the R Studio Version 2023.06.1+524 (2023.06.1+524) interface [21–23].

A statistically significant 95% CI band is one that would exclude the possibility of a stable or flat level of variation in the mean at that frequency. Therefore, a 95% CI band that cannot fit a horizontal line within the band has a periodic mean variation that is statistically different than zero. R^2^ values were calculated between each PC and the original data to see the proportion of variation explained by each principal component.

## Results

Following applications of VBPBB and GSBB, VBPBB and GSBB found one significant PC component in the Los Angeles NO_2_ levels. The annual PC component was significant and the other seven tested frequencies were not, as seen in Table 1. This table includes the 95% CI for the highest and lowest value in each band to show the points of greatest variation from the mean.

**Table 1:** VBPBB and GSBB 95% confidence intervals for the smallest and largest values in each confidence band.

	Frequency (1/ hours)	95% CI band minimum range VBPBB	95% CI band maximum range VBPBB	95% CI band minimum range GSBB	95% CI band maximum range GSBB	Median ratio of GSBB/VBPBB
Annual	1/8760	**(-6.878, -1.598)**	**(1.365, 10.894)**	**(-15.271, -5.971)**	**(3.629, 30.629)**	**3.630**
Annual harmonic	2/8760	(-1.843, 0.491)	(-0.405. 1.270)	(-15.471, 7.720)	(-9.371, 23.129)	8.184
Biweekly	1/400	(-0.545, 0.343)	(-0.348, 0.542)	(-15.971, 18.574)	(-16.571, 27.857)	27.417
Weekly	1/168	(-1.238, 0.364)	(-0.363, 1.183)	(-15.776, 20.929)	(-15.971, 37.129)	23.025
Weekly harmonic	2/168	(-0.761, 0.073)	(-0.071, 0.892)	(-16.076, 21.229)	(-15.373, 27.739)	55.643
Daily	1/24	(-0.927, 0.186)	(-0.186, 0.918)	(-16.421, 26.134)	(-15.471, 26.632)	4.316
Daily harmonic	2/24	(-0.802, 0.190)	(-0.193, 0.794)	(-16.672, 22.439)	(-15.622, 28.332)	41.880
Daily third harmonic	3/24	(-0.234, 0.051)	(-0.052, 0.233)	(-16.872, 22.330)	(-15.473, 26.937)	118.960

Note: **Bold results are significant P < 0.05**

GSBB was unable to identify any other significant PC components, however several VBPBB components were close to significant. The R^2^ value for the annual component explained 14.9% of the variation in the observed NO_2_ values. All other components tested accounted for less than 1% of the variation each, and approximately 1.7% in total.

Since there is not as yet a standardized procedure for picking the value of m and k we assessed multiple values as part of our analysis. M can range from 1 to n, the length of the data set. Using m = 1 means that no moving average is being applied and using high values of m closer to m = n means that there is more error introduced at the ends of the data series since a large number of values not averaged in at the ends for the points closer than m away from either end of the data string. We tested different values of m ranging from 49 to 17,251 but since it appeared to have a minimal effect on outcomes, and never affected significance, we kept our m’s to 2p + 1.

We tested values of k ranging from 1 to 8 but as they did not change the significance of the results we used k = 2 in our analysis. Generally, increasing k narrows the resultant energy transfer function and with our data tended to increase the amplitude of the calculated confidence bands. Increasing k smooths the resultant confidence bands and because it is iterated has the effect of making the highs higher and lows lower. Since increasing k is more computationally intensive we chose to keep k at 2 to increase efficiency.

### Annual periodic component

**Fig 3:**
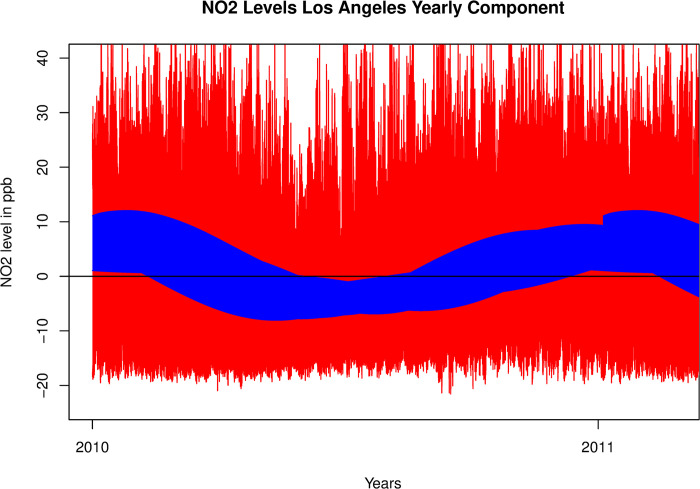
95% confidence bands for annual component calculated with GSBB and VBPBB.

The periodic mean variation for the yearly component was significantly different from zero for the VBPBB confidence bands as there were places where the 95% confidence band was completely below or above the horizontal line representing zero difference from the mean. This showed mean NO_2_ levels varied throughout the year with higher reported readings in the winter and lower in the summer. However, the GSBB confidence interval was much wider and although it excluded zero 0.1% of the time it was difficult to discern a seasonal pattern to the significant variations since the percentage of significant results was so low. This variation can be seen in Fig 3 where the blue confidence band moves completely above and below the horizontal line representing the mean NO_2_ reading. The red band represents the CI’s calculated through the GSBB process.

The median confidence interval with GSBB is 3.63 times larger than with VBPBB with most confidence intervals ranging from 2.13 to 7.05 times larger, showing that VBPBB is a superior method. In this case annual results from both methods are significant, however VBPBB shows a stronger result. 17.9% of the confidence intervals calculated as part of the confidence band exclude zero, while 0.1% of those using GSBB do, making it harder to observe a clear seasonal pattern. It is worthwhile to note the more consistently smooth CI band of VBPBB since it comes from the filtered data, illustrating that bandpass filtering has suppressed interfering frequencies.

The annual second harmonic, biweekly, weekly, daily components, and their harmonics were all found to be insignificant at the 95% CI level seen in Table 1. Each of these components produced VBPBB and GSBB 95% CI bands that could not exclude the possibility of zero variation in the mean at the given frequency. However, the 95% CI band for GSBB was routinely larger than that produced by VBPBB, ranging from just over 3.6 to nearly 119 times larger. The weekly harmonic and daily third harmonic components were close to significant for VBPBB and may warrant additional research concerning the arguments used to bandpass filter those frequencies.

## Discussion

In this analysis, VBPBB was used to isolate frequencies of interest and model principal components for nitrogen dioxide levels measured in Los Angeles. Results were compared to those produced by existing periodic bootstrap methods such as the GSBB. VBPBB was able to find evidence of one significant annual component while confidence bands produced by GSBB were substantially wider and therefore GSBB was not able to clearly distinguish a significant periodic variation.

Other PC components that were investigated were not significant, however more research is needed to determine optimal levels for KZFT bandpass filter arguments k and m, which may refine results. While VBPBB excludes interfering frequencies prior to block bootstrapping, and generally outperforms GSBB, it is possible that a different and more optimal choice of arguments for the KZFT bandpass filter may further improve results. This may lead to additional revelations about these other PC components.

The choice of arguments for the KZFT filters used for VBPBB is a limitation for this method since there is not currently a systematic way to optimize the levels for k and m. Increasing m decreases the size of the confidence interval bands. It also leads to increased errors in estimates due to incompletely applying the bandpass filter near the ends of the series. This could be a serious problem in smaller time series where the area near the ends makes up a larger proportion of the data. Changes in k move the filter from a simple moving average to approaching a Gaussian distribution.

It is possible that confounding factors such as changes in temperature or wind direction could also impact nitrogen dioxide levels and these variables were not accounted for in our analysis. The period studied also includes the Covid-19 shutdowns which affected work and commuting patterns. However, one of the strengths of VSPBB is that it allows variations caused by noise to be largely filtered out if they occur at a different frequency from any of the other principal components and so these factors are unlikely to have substantially impacted the observed bootstrapped results. There was substantially more variability in the weekly frequencies than in the daily or yearly periodograms. This could be due to variation in work weeks due to state or national holidays or seasonal travel patterns and may also have affected the significance of the data.

Better understanding of pollution patterns will aid in pollution reduction efforts by allowing us to pinpoint times of highest risk and direct mitigation efforts where they will have the greatest impact. This technique could be applied to other time series data with potential periodic correlation such as temperature or precipitation patterns, or to periodic data in other fields such as finance or epidemiology.
